# Review of the Bat Flies of Honduras, Central America (Diptera: Streblidae)

**DOI:** 10.1155/2013/437696

**Published:** 2013-03-24

**Authors:** Carl W. Dick

**Affiliations:** ^1^Department of Biology, Western Kentucky University, Bowling Green, KY 42101, USA; ^2^Department of Zoology, Field Museum of Natural History, Chicago, IL 60605, USA

## Abstract

Streblid bat flies are obligate and host-specific blood-feeding ectoparasites of bats. While the bat flies of some American countries are well studied (e.g., Panama, Venezuela), little is known about Honduran Streblidae. Accumulation of substantial numbers of specimens, from several different collections, has enabled a relatively thorough treatment of the fauna. This study is based on 2,236 specimens representing 17 genera and 43 species of Streblidae. Of those presently reported, 11 genera and 32 species are new records for Honduras, increasing the number of known genera and species by 65% and 74%, respectively. Collection and host data are listed for all known Honduran streblid bat fly species. Comments regarding host associations and specificity, geographic distribution, and taxonomic problems are given in the species accounts.

## 1. Introduction

Streblid bat flies are a worldwide group of obligate, blood-feeding ectoparasites of bats. The species tend to be host specific, with one fly species typically parasitizing one host species or a few closely related host species [1–3]. Some members of the streblid subfamily Nycterophiliinae are rather less host specific, often parasitizing two or more genera of hosts [4]. Distributional evidence suggests that they cospeciate with host species of bat, producing parallel phylogenies of host and parasite [5]. Streblids are often absent from bat species that roost solitarily or in temporary roosts [6]. Conversely, bat fly richness per host species seems generally to increase with roost size, duration, and the number of bats roosting there [7, 8]. The dynamics associated with bat roosts as they pertain to the biology, ecology, and host specificity among streblid bat flies is a critically understudied area. Perhaps attributing to the fact that bat host species often harbor more than one species of Streblidae is the observation that some bat flies prefer particular areas (microhabitats, e.g., fur or wing membranes) on the host's body [1, 9–11].

The taxonomy of Neotropical streblid genera relies primarily on overall body form, the presence or absence of a ctenidium along the posteroventral margin of the head, gross characteristics of the wings, leg chaetotaxy, and details of the thorax, especially the mesonotal and transverse sutures, and male reproductive structures. Generic identification can always be made using a stereozoom microscope. Species-level identifications may often be made using low magnification, but some species (especially *Trichobius* spp.) are best identified using slide-mounted specimens examined using a high-power compound microscope. A simplified key to New World genera is available in [12]. Other generic keys are available in [4, 13, 14]. The single best key to most New World streblid species is [4]. For species-level keys, see [13, 15–19]. Guerrero listed characteristics for 36 species of *Trichobius *[16].

Few Streblidae have been recorded previously from Honduras, largely due to the lack of ectoparasite sampling in the country. Honduran specimens have been previously reported in three publications. Wenzel et al. [1] reported *Strebla guajiro* (García & Casal) (as *S. carolliae* Wenzel) and *S. wiedemanni *Kolenati (as *S. vespertilionis *(Fabricius)). Wenzel [20] reported 12 species from Honduras but provided no data on specific records. Seven of the species are represented by specimens housed at the Field Museum of Natural History, Chicago (FMNH), and are considered valid records: *Trichobius costalimai *Guimaraes, *T. parasiticus* Gervais, *Trichobioides perspicillatus *(Pessôa & Galvão), *Megistopoda aranea* (Coquillett), *M. proxima *(Séguy), *Exastinion clovesi *(Pessôa & Guimarães), and *Strebla hertigi* Wenzel. The remaining five represent species whose presence in Honduras was inferred via geographical ranges [20]. These species included *Trichobius dugesioides *Wenzel, *T. joblingi* Wenzel, *T. uniformis* Curran, *Paratrichobius longicrus* (Miranda Ribeiro), and *Speiseria ambigua* Kessel. Peterson and Hůrka [21] reported *Trichobius intermedius* Peterson & Hůrka. Finally, Wenzel [4] reported *Anatrichobius scorzai* Wenzel. Incidentally, four of the five species inferred to occur in Honduras [20] are documented in the present specimen collection. Thus, six genera and 11 species of Honduran streblids had been documented by specimens prior to this publication. With the few exceptions noted, all known Honduran streblid species are represented by specimens housed at FMNH.

Moreover, there are few published records for Streblidae from countries that neighbor Honduras, such as Mexico, Belize, Nicaragua, and El Salvador. Dick [22] provided a generic level treatment for the bat flies of Guatemala. Wenzel compiled an unpublished catalog of the Streblidae of El Salvador (R. L. Wenzel, pers. comm.). Many records of Costa Rican streblids are known, yet these are unpublished as well. The nearest well-known streblid fauna is that of Panama [1], wherein 66 species of Panamanian Streblidae were treated. Guerrero's [23] compilation listed Wenzel et al.'s [1] species, plus three more for a total of 69 species for Panama.

## 2. Materials and Methods

The bulk of the specimens treated in this paper were collected by the author during 2001, as part of a survey project led by R. D. Bradley of Texas Tech University and R. A. Van Den Bussche and M. Hamilton of Oklahoma State University. During this expedition, 964 bats were collected at 15 localities. Bats were collected with nylon mist nets and by hand and usually kept individually in cloth bags. Because ectoparasite collection was not the main focus of the expedition, not all captured bats were sampled for parasites, and there were instances when cross-host contamination of parasites was likely. These instances are noted in the species accounts and addressed in the discussion. Bats were anesthetized with ethyl ether and brushed for ectoparasites, which were stored in vials of 95% ethanol. Streblid flies were collected from 242 individual bats. Most of the flies were studied under a dissection microscope. Others were slide-mounted in Canada balsam for examination under a compound microscope. Confirmations of identifications were made by comparison of the Honduran specimens to type specimens and other reference collections housed at the FMNH.

**Figure 1:**
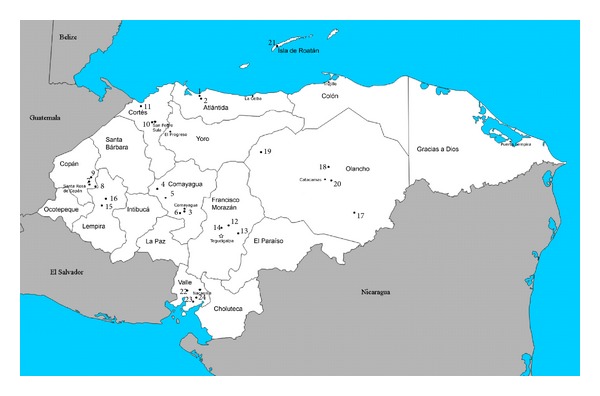
Map of Honduran bat fly collection localities. Locality numbers (numbered closed circles) correspond to specific localities in Gazetteer (Table 1).

Specimens and specimen records for this project came from three primary sources. The TTU Honduras expedition yielded 381 records and 2051 specimens. Additionally, 47 records and 141 specimens were provided by T. J. McCarthy and R. P. Eckerlin, collected during the project “Mastozoolgía en el Núcleo de Centroamerica” (MANCA). Eighteen records and 44 specimens from the FMNH, representing new and previously reported specimens, were studied. Previously reported specimens (e.g., [1, 4, 20, 21]) were reexamined. Fly identifications were made by the author, but historical FMNH specimens were originally identified by R. L. Wenzel. Thus, a total of 446 records and 2,236 specimens of Streblidae were examined as the basis of this paper. Most fly specimens have been deposited in the FMNH, but representative MANCA specimens were deposited at Carnegie Museum (CM), Pittsburgh, PA, USA, and a synoptic collection was deposited at TTU. Bat host vouchers exist for most of the parasite records; all flies collected during the TTU project can be traced to host bat specimens in the mammal collections of TTU. Flies collected during the MANCA project can be traced to bat specimens housed at CM or the American Museum of Natural History (AMNH), New York. Nomenclature used for bats generally follows Simmons [24]. The accounts below provide an overview of each streblid genus known from Honduras, presented by subfamily. Species accounts list streblid species and primary reference, records from Honduras, and comments when relevant. A concise list of all streblid species known from Honduras appears in Appendix A. A list of Honduran bat fly host associations is presented in Appendix B. A gazetteer of collection localities is presented in Table 1, which corresponds to the map of collection localities (Figure 1). Unless indicated otherwise, fly specimens were collected by the author.

## 3. Species Accounts

### 3.1. Family Streblidae Kolenati, 1863

#### 3.1.1. Subfamily Nycterophiliinae Wenzel, 1966 

*Genus Nycterophilia Ferris, 1916*

 
*Nycterophilia* Ferris, 1916:436.

This genus comprises five described species: *N. coxata* Ferris, *N. fairchildi* Wenzel, *N. mormoopsis* Wenzel, *N. natali* Wenzel, and *N. parnelli* Wenzel. Only *N. coxata* is known to occur in Honduras. Species in this genus are associated with bats of the families Mormoopidae, Natalidae, and Phyllostomidae (*Leptonycteris* Lydekker). Species of this genus are strongly compressed laterally, resembling fleas. The species are adapted to live inside the hair of their hosts [25].

*Nycterophilia coxata Ferris*

 
*Nycterophilia coxata* Ferris, 1916:437. Plate 22, Figures 5-6.

*Honduran Records *(*362 Specimens*)

 COMAYAGUA: f1 from *Sturnira lilium* (E. Geoffroy), 4.8 km N, 8.7 km W Comayagua (Playitas), 10 July 2001.
 COPÁN: m193, f164, 1 sex undetermined from *Pteronotus parnellii* (Gray), 20 km SE Santa Rosa de Copán, 17 July 2001.
 OLANCHO: m1, f2 from *P. parnellii*, 4 km E Catacamas (Escuela de Sembrador), 20 July 2001.

In Honduras, the vast majority (99.7%) of *N. coxata* specimens were associated with the mormoopid bat *P. parnellii*. The single specimen from *S. lilium* is regarded as a contaminant, as a specimen of *P. gymnonotus* Natterer was collected on the same day. 

#### 3.1.2. Subfamily Streblinae Speiser, 1900

*Genus Anastrebla Wenzel, 1966*

 
*Anastrebla* Wenzel, 1966:627.

This genus comprises five described species: *A. caudiferae* Wenzel, *A. mattadeni* Wenzel, *A. modestini *Wenzel, *A. nycteridis* Wenzel, and *A. spurrelli* Wenzel. Only *A. modestini* is known to occur in Honduras. Species of *Anastrebla* are typically associated with phyllostomid bats of the subfamily Glossophaginae.

*Anastrebla modestini Wenzel*

 
*Anastrebla modestini* Wenzel, 1966:629. Figures 138A, 139C, D.

*Honduran Record *(*1 Specimen*)

 FRANCISCO MORAZÁN: f1 from *Glossophaga soricina* (Pallas), Parque Zoológico El Picacho, 5 July 2001.

Elsewhere (e.g., Panama and Venezuela [1, 4]), this species is associated with *Anoura geoffroyi* Gray. Thus, its association with *G. soricina* is puzzling. Only one *A. geoffroyi* was collected on 18 July 2001.

*Genus Metelasmus Coquillett 1859*

 
*Metelasmus* Coquillett, 1907:292.

This genus comprises two species, *Metelasmus pseudopterus* and *M. wenzeli* Graciolli & Dick, and only the former is known from Honduras. *Metelasmus pseudopterus* parasitizes certain species of large *Artibeus *Leach, including *A. jamaicensis *Leach*, A. fimbriatus *Gray, and* A. planirostri*s Spix. *Metelasmus wenzeli* parasitizes *Sturnira lilium* in eastern Paraguay and southern Brazil. An undescribed species, similar to *M. wenzeli*, is known from Guatemalan *Sturnira* sp. It is probable that this undescribed species also occurs in Honduras. Species of *Metelasmus* are the only vestigially winged members of the subfamily Streblinae. The species are dorsoventrally compressed and possess a ctenidium of rearward facing spines on the posteroventral margin of the head. The species appear to be adapted to live within the fur of their hosts [24].

*Metelasmus pseudopterus Coquillett*

 
*Metelasmus pseudopterus* Coquillett, 1907:292.

*Honduran Records *(*4 Specimens*)

 ATLÁNTIDA: m1, f1 from *Artibeus jamaicensis*, Jardin Botanico Lancetilla, 12 July 2001; f1 from *Carollia sowelli* Baker et al., Jardin Botanico Lancetilla, 12 July 2001; m1 from *A. lituratus* (Olfers), Jardin Botanico Lancetilla, 14 July 2001.

This species is a characteristic parasite of *Artibeus jamaicensis* in the Central and northern South America and of *A. planirostris *and *A. fimbriatus* in southern South America. Graciolli and Dick [26] discussed its association with *A. lituratus*.

*Genus Paraeuctenodes Pessôa and Guimarães,* *1937 *

 
*Paraeuctenodes* Pessôa & Guimarães, 1937:257.

Two described species belong to this genus, *Paraeuctenodes longipes* Pessôa & Guimarães and *P. similis* Wenzel, and only the former is known from Honduras. In Honduras and Venezuela [4], the primary host species of *P. longipes* are *Glossophaga* spp., while the primary host of *P. similis* is *Carollia perspicillata *(Linnaeus). Both species of *Paraeuctenodes *are dorsoventrally flattened and possess a ctenidium of rearward facing spines on the posteroventral margin of the head. These characteristics appear to adapt the species to live within the fur of their hosts [25].

*Paraeuctenodes longipes Pessôa and Guimarães*

 
*Paraeuctenodes longipes* Pessôa and Guimarães, 1937:258.

*Honduran Records *(*3 Specimens*)

 COMAYAGUA: f1 from *Glossophaga commissarisi* Gardner, 4.8 km N, 8.7 km W Comayagua (Playitas), 10 July 2001.
 OLANCHO: m1, f1 from *G. soricina*, 4 km E Catacamas (Escuela de Sembrador), 20 July 2001.

*Genus Strebla Wiedemann 1824*

 
*Strebla* Wiedemann, 1824:19.

The genus *Strebla* comprises 25 described species and is second only to the genus *Trichobius* in number of species. Seven species of this genus are known to occur in Honduras. Species of *Strebla* are dorsoventrally flattened and possess a ctenidium of rearward facing spines on posteroventral margin of the head. This form appears to adapt species of *Strebla *to live inside the fur of the host [25].

*Strebla chrotopteri Wenzel*

 
*Strebla chrotopteri* Wenzel, 1976:144, Figures 60H, 64E.

*Honduran Records* (*5 Specimens*)

 LEMPIRA: m2 from *Chrotopterus auritus* (Peters), Parque Nacional Celaque, Centro de Visitantes, 11 October 1992, T. J. McCarthy, leg.; m2, f1 from *C. auritus*, Parque Nacional Celaque, Centro de Visitantes, 11 October 1992, J. V. Planz, leg.

This species is known only from the phyllostomine bat *C. auritus*.

*Strebla curvata Wenzel*

 
*Strebla curvata* Wenzel, 1976:148, Figures 60D, 63F.

*Honduran Records *(*7 Specimens*)

 ATLÁNTIDA: f1 from *Artibeus lituratus*, Jardin Botanico Lancetilla, 12 July 2001; m2 from *Glossophaga soricina*, Jardin Botanico Lancetilla, 13 July 2001.
 COMAYAGUA: f2 from *G. soricina*, 4.8 km N, 8.7 km W Comayagua (Playitas), 10 July 2001.
 OLANCHO: f1 from *G. soricina*, 3 km N Catacamas (Escuela Nacional de Agricultura), 20 July 2001; f1 from *G. soricina*, 4 km E Catacamas (Escuela de Sembrador), 20 July 2001.

*Strebla curvata* is a characteristic parasite of *Glossophaga* spp., particularly *G. soricina*, in both Honduras and Venezuela [4]. The species is remarkably similar to a congener, *S. guajiro*, which is a characteristic parasite of species of *Carollia* Gray.

*Strebla diphyllae Wenzel*

 
*Strebla diphyllae* Wenzel, 1966:613, Figures 124C, 133.

*Honduran Records *(*2 Specimens*)

 ATLÁNTIDA: m1, f1 from *Diphylla ecaudata*, Lancetilla, 9 August 1969, R. K. LaVal, leg.

*Strebla guajiro* (*García & Casal*)

 
*Euctenodes guajiro* García & Casal, 1965:14, Figures 10-16.

*Honduran Records *(*13 Specimens*)

 ATLÁNTIDA: f1 from *Artibeus lituratus*, Jardin Botanico Lancetilla, 12 July 2001; m1 from *Carollia perspicillata*, Jardin Botanico Lancetilla, 12 July 2001; m3 from *C. sowelli*, Jardin Botanico Lancetilla, 12 July 2001; m1, f2 from *C. sowelli*, Jardin Botanico Lancetilla, 14 July 2001.
 LEMPIRA: m2 from *C. brevicauda* (Schinz), Parque Nacional Celaque, Centro de Visitantes, 11 October 1992, J. V. Planz, leg.; f1 from *C. brevicauda*, Parque Nacional Celaque, Centro de Visitantes, 17 February 1998, R. P. Eckerlin, leg.
 OLANCHO: m1 from *Glossophaga soricina*, 3 km N Catacamas (Escuela Nacional de Agricultura), 20 July 2001.
 ISLA ROATAN: m1 from unknown host, Coxen Hole, 9 January 1940, D. D. Davis, leg. (as *S. carolliae* Wenzel [1]).

The characteristic hosts of *S. guajiro* in Honduras were species of *Carollia*. In Venezuela, 98.5% of the 586 specimens collected there were taken from *Carollia* spp., and most of these were from *C. perspicillata* [4]. The single specimen from *A. lituratus* is regarded as a contaminant, as specimens of *C. perspicillata* were collected on the same day.

*Strebla hertigi Wenzel*

 
*Strebla hertigi *Wenzel, 1966:596. Figures 122B, 125F, 127B.

*Honduran Records *(*5 Specimens*)

 FRANCISCO MORAZON: m2, f2 from *Phyllostomus discolor* Wagner, 12 mi N Tegucigalpa, no date, “GHJ”, leg. ([20]; data compiled by author).
 VALLE: m1 from *P. discolor*, 3 km N, 12.5 km SW San Lorenzo, 7 July 2001.

Throughout its range, *Strebla hertigi* is a characteristic parasite of *P. discolor*. Within northern portions of its range, this species cooccurs with a congener, *S. mirabilis*, on the host species *P. hastatus *(Pallas). However, numerical dominance of each species varied depending on latitude [27]. No *P. hastatus* were collected during the present study, and no *S. mirabilis* were reported from any host species. Wenzel et al. [1] and Wenzel and Tipton [27] discussed the issue of competitive displacement for these species of *Strebla *on the host bats *P. discolor* and *P. hastatus*.

*Strebla galindoi Wenzel*

 
*Strebla galindoi *Wenzel, 1966:604. Figures 123D, 124F, 125D, 130.

*Honduran Records *(*1 Specimen*)

 ATLÁNTIDA: m1 from *Tonatia saurophila* Koopman & Williams, Jardin Botanico Lancetilla, 12 July 2001.

*Strebla kohlsi Wenzel*

 
*Strebla kohlsi *Wenzel, 1966:618. Figure 123C.

*Honduran Records *(*5 Specimens*)

 ATLÁNTIDA: m3, f2 from *Lophostoma silvicolum* d'Orbigny, Jardin Botanico Lancetilla, 14 July 2001.

*Strebla wiedemanni Kolenati*

 
*Hippobosca vespertilionis* Fabricius, 1805:339. Suppressed by the ICZN, 1936:29.
 
*Strebla wiedemannii* Kolenati, 1856:46.

*Honduran Records *(*69 Specimens*)

 ATLÁNTIDA: m1, f1 from *Desmodus rotundus* (E. Geoffroy), Lancetilla, 1 August 1969, R. Valdez, leg. (as *S. vespertilionis* (Fabricius) [1]; data compiled by author); m25, f17 from *D. rotundus*, Jardin Botanico Lancetilla, 12 July 2001; m11, f7 from *D. rotundus*, Jardin Botanico Lancetilla, 15 July 2001.
 COPÁN: m2 from *D. rotundus*, no specific locality, May 1938, M. Ennis, leg. (as *S. vespertilionis* [1]; data compiled by author).
 FRANCISCO MORAZON: m1 from *D. rotundus*, Parque Nacional La Tigra, 6 July 2001.
 LEMPIRA: f1 from *D. rotundus*, 3.5 mi N Gracias, 8 January 1938, P. O. McGrew, leg. (as *S. vespertilionis* [1]; data compiled by author); m2 from *D. rotundus*, Parque Nacional Celaque, Centro de Visitantes, 11 October 1992, J. V. Planz, leg.
 VALLE: f1 from *D. rotundus*, 13 km W, 3 km S Nacaome, 9 July 2001.

The characteristic host of *Strebla wiedemanni* in Honduras and elsewhere in the New World is the common vampire bat *Desmodus rotundus*.

#### 3.1.3. Subfamily Trichobinae Jobling, 1936

*Genus Anatrichobius Wenzel, 1966*

 
*Anatrichobius *Wenzel, 1966:502.

This genus comprises two described species, *Anatrichobius scorzai* Wenzel and *A. passosi* Graciolli, only the former of which occurs in Honduras. These species are among the few New World streblids associated with vespertilionid bats; *Anatrichobius* spp. are restricted to species of the genus *Myotis *Kaup and appear restricted to elevations from 600 to 1900 m [28].

*Anatrichobius scorzai Wenzel*

 
*Anatrichobius scorzai* Wenzel, 1966:503. Figures 76–78.

*Honduran Records *(*6 Specimens*)

 FRANCISCO MORAZÁN: m1, f1 from *Myotis keaysi* J. A. Allen (RKL 2495-2521), 1 km W Talanga, 26 July 1969, R. K. LaVal, leg. [4].
 OLANCHO: m3, f1 from *M. keaysi* (CM 118609), Parque Nacional de La Muralla, Los Cuatro Pavos, 18 October 1992, T. J. McCarthy, leg.

Elevational data for these records are not available, but they were likely collected in the Honduran highlands. The elevation of Talanga is approximately 840 m, while Parque Nacional de La Muralla is a montane reserve, the highest point being 1,986 m.

*Genus Aspidoptera Coquillett, 1899*

 
*Aspidoptera* Coquillett, 1899:334.

This genus comprises three species, *Aspidoptera delatorrei *Wenzel*, A. falcata,* and *A. phyllostomatis* [4, 23]; the latter two occur in Honduras. Species of this genus are restricted to phyllostomid bats of the subfamily Stenodermatinae.

*Aspidoptera falcata Wenzel*

 
*Aspidoptera falcata* Wenzel, 1976:104, Figure 42A.

*Honduran Records *(*39 Specimens*)

 ATLÁNTIDA: m1, f1 from *Sturnira lilium*, Jardin Botanico Lancetilla, 12 July 2001.
 COMAYAGUA: m9, f7 from *S. lilium*, 4.8 km N, 8.7 km W Comayagua (Playitas), 10 July 2001.
 LEMPIRA: m1 from *S. ludovici* Anthony, Parque Nacional Celaque, Centro de Visitantes, 11 October, 1992, T. J. McCarthy, leg.; m2 from *S. ludovici*, Parque Nacional Celaque, Centro de Visitantes, 12 October 1992, T. J. McCarthy, leg.; m1 from *S. ludovici*, Parque Nacional Celaque, Don Tomas, 12 February 1998, R. P. Eckerlin, leg.; f1 from *S. ludovici*, Parque Nacional Celaque, Don Tomas, 13 February 1998, R. P. Eckerlin, leg.; m7, f3 from *S. ludovici*, Parque Nacional Celaque, Centro de Visitantes, 17 February 1998, R. P. Eckerlin, leg.
 OLANCHO: f1 from *Noctilio leporinus *(Linnaeus), 4 km E Catacamas (Escuela de Sembrador), 17 July 2001; m2, f1 from *S. lilium*, 4 km E Catacamas (Escuela de Sembrador), 19 July 2001; f1 from *S. lilium*, 3 km N Catacamas (Escuela Nacional de Agricultura), 20 July 2001.
 VALLE: f1 from *S. lilium*, 13 km W, 3 km S Nacaome, 10 July 2001.

*Aspidoptera falcata* is a characteristic parasite of several species of *Sturnira* Gray, including *S. lilium*, *S. ludovici*, and *S. tildae* de la Torre. In Venezuela, 99.0% of the 755 *A. falcata* specimens collected there were taken from these three species of *Sturnira *[4]. The record from the fishing bat *Noctilio leporinus* almost certainly resulted from sampling contamination. *Aspidoptera falcata* is morphologically very similar to *A. delatorrei* and can be positively identified only by examining the postgonites (falciform in *A. falcata*).

*Aspidoptera phyllostomatis *(*Perty*)

 
*Lipoptena phyllostomatis* Perty, 1833:190, Figure 17, Plate 37.

*Honduran Records *(*14 Specimens*)

 ATLÁNTIDA: m5, f5 from *Artibeus jamaicensis*, Jardin Botanico Lancetilla, 12 July 2001; f1 from *Glossophaga soricina*, Jardin Botanico Lancetilla, 13 July 2001; m1, f2 from *A. lituratus*, Jardin Botanico Lancetilla, 13 July 2001.

The primary hosts of *Aspidoptera phyllostomatis* in Paraguay were *Artibeus fimbriatus* and *A. planirostris*, collectively hosting 93.1% of the 29 specimens. The remaining 2 of 29 (6.9%) specimens were collected from 2 individuals of *A. lituratus*. The association between *Aspidoptera phyllostomatis* and *Artibeus lituratus* may be real, albeit rare. In Venezuela, 95.5% of *Aspidoptera phyllostomatis* were associated with *Artibeus jamaicensis/planirostris*, but Wenzel [4] did not consider *A. lituratus* to be a host of this fly species.

*Genus Exastinion Wenzel, 1966*

 
*Exastinion* Wenzel, 1966:558.

This genus comprises three species, *Exastinion clovisi* (Pessôa & Guimarães), *E. deceptivum* Wenzel, and *E. oculatum* Wenzel, of which only the former is known to occur in Honduras. All species in this genus parasitize species of *Anoura* Gray. Both *E. clovisi* and *E. deceptivum* occur on *A. geoffroyi*, but in some locations (e.g., Manu, Peru) the former species parasitizes hosts from lower elevations (1000–1920 m) while the latter species parasitizes hosts from higher elevations (1920–4137 m). There are to my knowledge no instances of cooccurrence of these species on the same individual bat (C. W. Dick, unpublished data).

*Exastinion clovisi* (*Pessôa & Guimarães*)

 
*Aspidoptera clovisi* (Pessôa & Guimarães), 1937:262. Figures 5-6.

*Honduran Records *(*5 Specimens*)

 COMAYAGUA: m1, f2 from *Anoura geoffroyi*, Siguatepeque, 18 July 2001.
 FRANCISCO MORAZÁN: m1, f1 from *A. geoffroyi*, 12 mi N Tegucigalpa, 8 June 1963, D. C. Carter, leg. ([20]; data compiled by author).

In Venezuela, 98.8% of the 340 *E. clovisi* were collected from *A. geoffroyi*, *A. latidens* Handley, and *A. caudifer* (E. Geoffroy), in descending order.

*Genus Mastoptera Wenzel, 1966*

 
*Mastoptera* Wenzel, 1966:512.

This genus comprises two described species, *Mastoptera guimaraesi* Wenzel and *M. minuta *(Costa Lima), and appears to be restricted to phyllostomid bats of the subfamily Phyllostominae. This genus contains the smallest of New World streblid species, with some specimens of *M. minuta* measuring only 0.73 mm in total length [12]. The genus is in need of revision [4].

*Mastoptera guimaraesi Wenzel*

 
*Mastoptera guimaraesi* Wenzel, 1966: 514, Figures 82C, 83, 84.

*Honduran Records *(*3 Specimens*)

 ATLÁNTIDA: m1, f2 from *Phyllostomus hastatus*, Lancetilla, 9 August 1969, R. Valdez & R. K. LaVal, leg.

In Panama [1] and Venezuela [4], the characteristic host of this species is *Phyllostomus hastatus*.

*Mastoptera minuta *(*Costa Lima*)

 
*Aspidoptera minuta* Costa Lima, 1921:21, Figure 2, Plate 2.

*Honduran Records *(*5 Specimens*)

 ATLÁNTIDA: m4, f1 from *Lophostoma silvicolum*, Jardin Botanico Lancetilla, 14 July 2001.

In Venezuela, the characteristic host of *M. minuta* is *Lophostoma silvicolum* (d'Orbigny) [4]. The taxonomy of *Mastoptera* species is complex and poorly understood, and Wenzel [4] noted that there were undescribed species within *M. minuta*.

*Mastoptera sp. *(*Minuta Complex*)

*Honduran Records *(*23 Specimens*)

 ATLÁNTIDA: m14, f9 from *Lophostoma brasiliense* Peters, Jardin Botanico Lancetilla, 13 July 2001.

The 23 Honduran specimens were collected from one host individual of *L. brasiliense*. The taxonomy of *Mastoptera* species is complex and poorly understood, and Wenzel [4] noted that there were undescribed species within *M. minuta*. Here I refer specimens to *M. minuta* species complex. The group is in need of revision.

*Genus Megistopoda Macquart, 1852*

 
*Megistopoda* Macquart, 1852:332.

This genus comprises three described species: *Megistopoda aranea, M. proxima, and M. theodori* [4]. Wenzel [4] noted that the taxonomy of this genus is confused and in need of revision, as there are undescribed species within this genus. Wenzel [4] questioned the distinctness of *M. theodori* and stated that it might be synonymous with *M. proxima*. I consider the two species to be distinct (see *M. theodori* account below), but note that the *proxima* group of species contains several undescribed species. *Megistopoda *of the *aranea *type possess extremely elongated hind legs and very narrow wings and parasitize species of *Artibeus *(but not *Dermanura* spp.). *Megistopoda *of the *proxima* type (including *M. theodori*) possess less elongated hind legs and broader wings and parasitize species of *Sturnira*. However, recent specimens from western Ecuador document the existence of two other undescribed *aranea* type species, one from the west slope endemic *Artibeus fraterculus* Anthony and one from *Platyrrhinus dorsalis* (Thomas) (C. W. Dick, unpublished data). Collectively, species of *Megistopoda* are restricted to phyllostomid bats of the subfamily Stenodermatinae. All species possess elongated hind legs and a shield-like ventral thorax, which adapts them for movement over the fur of their hosts [25].

*Megistopoda aranea *(*Coquillett*)

 
*Pterellipsis aranea* Coquillett, 1899:344.

*Honduran Records *(*31 Specimens*)

 ATLÁNTIDA: m4, f2 from *Artibeus jamaicensis*, Jardin Botanico Lancetilla, 12 July 2001; m1 from *A. lituratus*, Jardin Botanico Lancetilla, 12 July 2001; m1 from *Glossophaga soricina*, Jardin Botanico Lancetilla, 15 July 2001.
 LEMPIRA: m5, f2 from *A. jamaicensis*, Parque Nacional Celaque, Centro de Visitantes, 17 February 1998, R. P. Eckerlin, leg.
 OLANCHO: m1 from *A. jamaicensis*, 4 km E Catacamas (Escuela de Sembrador), 19 July 2001; m1, f1 from *A. jamaicensis*, 4 km E Catacamas (Escuela de Sembrador), 20 July 2001; m1 from *A. jamaicensis*, 3 km E Catacamas (Escuela Nacional de Agricultura), 20 July 2001; m5, f2 from *A. intermedius *J. A. Allen, 3 km N Catacamas (Escuela Nacional de Agricultura), 20 July 2001.
 ISLA ROATAN: m2, f2 from *A. jamaicensis*, “west end of island,” 13 January 1994, R. P. Eckerlin, leg.
 Unidentified Location: f1 from *A. jamaicensis*, “Tapasuna”, 1 December–1 January 1937-1938, P. O. McGrew, leg. ([20]; data compiled by author)

This species is a stenoxenous parasite of certain species of large *Artibeus*. In Venezuela, 97% of the 546 specimens collected were associated with *A. jamaicensis/planirostris* [4]. In Paraguay, however, the primary hosts were *Artibeus fimbriatus* (70.2% of 104 specimens) and *A. planirostris* (27.9%).

*Megistopoda proxima *(*Séguy*)

 
*Pterellipsis proxima* Séguy, 1926:194, Figures 2–6.

*Honduran Records *(*58 Specimens*)

 ATLÁNTIDA: m1 from *Sturnira lilium*, Jardin Botanico Lancetilla, 12 July 2001; f1 from *Artibeus lituratus*, Jardin Botanico Lancetilla, 12 July 2001; m1, f1 from *A. lituratus*, Jardin Botanico Lancetilla, 14 July 2001; m1 from *Dermanura *(= *Artibeus*) *phaeotis* (Miller), Jardin Botanico Lancetilla, 13 July 2001.
 COMAYAGUA: m17, f13 from *S. lilium*, 4.8 km N, 8.7 km W Comayagua (Playitas), 10 July 2001; m2, 1 sex undetermined from *Glossophaga commissarisi*, 4.8 km N, 8.7 km W Comayagua (Playitas), 10 July 2001.
 OLANCHO: m2 from *S. lilium*, Danali, 78 mi ENE by E Rio Coco, 15 May 1963, D. C. Carter, leg. ([20]; data compiled by author); m2, f5 from *S. lilium*, 4 km E Catacamas (Escuela de Sembrador), 19 July 2001; f1 from *G. soricina*, 4 km E Catacamas (Escuela de Sembrador), 19 July 2001.
 VALLE: m6, f4 from *S. lilium*, 13 km W, 3 km S Nacaome, 10 July 2001.

The characteristic host of *M. proxima* in Paraguay was *S. lilium* (hosting 98.1% of 372 specimens) [29]. In Venezuela, all of the 965 specimens were from *S. lilium* [4], but specimens from hosts other than *S. lilium* were simply referred to the *M. proxima* complex. *Megistopoda proxima* as currently described represents a complex of species [4], and in general, the true *M. proxima* are specific to *S. lilium*. This group of bat flies has not been studied in detail.

*Megistopoda theodori Wenzel*

 
*Megistopoda theodori *Wenzel, 1966:545. Figure 100B.

*Honduran Records *(*52 Specimens*)

 ATLÁNTIDA: f1 from *Uroderma bilobatum* Peters, Jardin Botanico Lancetilla, 14 July 2001.
 FRANCISCO MORAZON: m10, f8 from *Sturnira ludovici*, Parque Nacional La Tigra, 6 July 2001.
 LEMPIRA: f3 from *S. ludovici*, Parque Nacional Celaque, Centro de Visitantes, 11 October 1992, T. J. McCarthy, leg.; m4, f2 from *S. ludovici*, Parque Nacional Celaque, Centro de Visitantes, 12 October 1992, T. J. McCarthy, leg.; m4, f1 from *S. ludovici*, Parque Nacional Celaque, Centro de Visitantes, 12 October 1992, J. V. Planz, leg.; f1 from *S. ludovici*, Parque Nacional Celaque, Centro de Visitantes, 13 October 1992, T. J. McCarthy, leg.; m3, f4 from *S. ludovici*, Parque Nacional Celaque, Don Tomas, 12 February 1998, R. P. Eckerlin, leg.; m1, f3 from *S. ludovici*, Parque Nacional Celaque, Don Tomas, 13 February 1998, R. P. Eckerlin, leg.; m2, f1 from *S. ludovici*, Parque Nacional Celaque, Centro de Visitantes, 17 February 1998, R. P. Eckerlin, leg.
 OLANCHO: m3, f1 from *S. ludovici*, Parque Nacional Agalta, Sendero a la Picucha, 7 March 1998, R. P. Eckerlin, leg.

Wenzel [4] debated the validity of *M. theodori* and stated that it may be synonymous with *M. proxima*. A final decision was deferred until further studies had been undertaken. Moreover, Wenzel [4] stated that flies currently referred to *M. proxima* represent a complex of closely related species and that each host species may indeed harbor a distinct species of *Megistopoda*. Recent study concurs with [4] in this regard. It is clear that a thorough revision of *Megistopoda* is needed in order to resolve these issues. Although the thorax shows marked lateral compression as in *M. proxima*, the dorsal thoracic plate is less humped. I refer specimens from *S. ludovici* to *M. theodori*.

*Genus Neotrichobius Wenzel & Aitken, 1966*

 
*Neotrichobius* Wenzel & Aitken, 1966:536.

Four described species belong to this genus and include *Neotrichobius bisetosus* Wenzel, *N. delicatus* Machado-Allison, *N. ectophyllae* Wenzel, and *N. stenopterus* Wenzel & Aitken. Only the last species is known from Honduras. An undescribed species of *Neotrichobius* has been collected from *Mesophylla macconnelli *Thomas in La Selva, Costa Rica [30], and in Ecuador (C. W. Dick, unpublished data). *Neotrichobius delicatus *may represent a complex of species [4]. *Neotrichobius* spp. are typically associated with phyllostomid bats of the subfamily Stenodermatinae and with *Rhinophylla pumilio *Peters (Rhinophyllinae).

*Neotrichobius stenopterus Wenzel & Aitken*

 
*Neotrichobius stenopterus* Wenzel & Aitken, 1966:539. Figures 97–99.

*Honduran Records *(*3 Specimens*)

 ATLÁNTIDA: f1 from *Dermanura* ( =  *Artibeus*) *toltecus *(Saussure), Jardin Botanico Lancetilla, 12 July 2001; m2 from *D. phaeotis*, Jardin Botanico Lancetilla, 13 July 2001.

*Genus Noctiliostrebla Wenzel 1966*

 
*Noctiliostrebla* Wenzel 1966:560.

This genus comprises four recognized species: *Noctiliostrebla aitkeni *Wenzel*, N. dubia *(Rudow)*, N. maai* Wenzel, and *N. traubi* Wenzel [4, 23]. Species of this genus are restricted to fishing or bulldog bats of the genus *Noctilio* Linnaeus (family Noctilionidae). Two species are known from *N. leporinus* and two from *N. albiventris* Desmarest, but based on data from Venezuela, the two species infesting each host species never cooccur on the same host individual [25]. *Noctiliostrebla* spp. possess vestigial wings and are rather similar in overall morphology.

*Noctiliostrebla traubi Wenzel*

 
*Noctiliostrebla traubi* Wenzel, 1966:565. Figures 106, 107B, D.

*Honduran Records *(*34 Specimens*)

 OLANCHO: m1 from *Noctilio leporinus*, 4 km E Catacamas (Escuela de Sembrador), 17 July 2001; m17, f16 from *N. leporinus*, 4 km E Catacamas (Escuela de Sembrador), 19 July 2001.

The characteristic host of *Noctiliostrebla traubi* in Honduras and elsewhere is the fishing bat *Noctilio leporinus* (Noctilionidae).

*Genus Paradyschiria Speiser, 1900*

 
*Paradyschiria* Speiser, 1900:55.

This genus comprises five species: *Paradyschiria curvata *Wenzel*, P. fusca *Speiser*, P. lineata *Kessel*, P. parvula* Falcoz, and *P. parvuloides* Wenzel [4, 23]. Species of this genus are wholly restricted to fishing or bulldog bats of the genus *Noctilio* (family Noctilionidae). Two species parasitize only *N. leporinus*, while three species parasitize only *N. albiventris*. As with *Noctiliostrebla *spp., species of Venezuelan *Paradyschiria* never appear to cooccur on the same host individual [25]. *Paradyschiria* spp. are the only wingless streblid bat flies.

*Paradyschiria fusca Speiser*

 
*Paradyschiria fusca* Speiser, 1900:56, Figure 1, Plate 3.

*Honduran Records *(*43 Specimens*)

 OLANCHO: m8, f5 from *Noctilio leporinus*, 4 km E Catacamas (Escuela de Sembrador), 17 July 2001; m13, f17 from *N. leporinus*, 4 km E Catacamas (Escuela de Sembrador), 19 July 2001.

The characteristic host of *P. fusca* in Honduras and elsewhere is the fishing bat *Noctilio leporinus*.

*Paradyschiria parvuloides Wenzel*

 
*Paradyschiria parvuloides* Wenzel, 1966: 575. Figures 110D, 112C, D, 113B, 114.

*Honduran Records *(*10 Specimens*)

 OLANCHO: m8, f2 from *Noctilio albiventris*, 4 km E Catacamas (Escuela de Sembrador), 19 July 2001.

The characteristic host of *P. parvula* in Honduras and elsewhere is *Noctilio albiventris*.

*Genus Paratrichobius Costa Lima, 1921*

 
*Paratrichobius* Costa Lima, 1921:20.

This genus comprises six described species, *Paratrichobius americanus *Peterson & Ross, *P. dunni *(Curran)*, P. longicrus *(Ribeiro)*, P. lowei *Wenzel*, P. salvini *Wenzel, and *P. sanchezi *Wenzel. All are known to be associated with bats of the phyllostomid subfamily Stenodermatinae. *Paratrichobius* spp. are fully winged but possess a shield-like ventral thorax very long hind legs; these characteristics appear to adapt these species to life in the fur and facilitate their evasive behavior of movement over the top of the fur. The taxonomy of this genus is confused and a revision is needed; in particular, the nominal species *P. longicrus* and *P. salvini *are most likely species complexes [4].

*Paratrichobius dunni *(*Curran*)

 
*Speiseria dunni* Curran, 1935:7, Figure 6.

*Honduran Records *(*3 Specimens*)

 ATLÁNTIDA: f1 from *Dermanura* ( =  *Artibeus*) *phaeotis*, Jardin Botanico Lancetilla, 13 July 2001; m1, f1 from *Uroderma bilobatum*, Jardin Botanico Lancetilla, 14 July 2001.

More than 98% of the 102 *P. dunni* collected in Venezuela were taken from *Uroderma bilobatum* and *U. magnirostrum* Davis [4]. Species of *Dermanura* host a unique species, *P. lowei*, and the present records from *D. phaeotis* are probably contaminants.

*Paratrichobius longicrus *(*Miranda Ribeiro*)

 
*Trichobius longicrus* Miranda Ribeiro, 1907:236, Plate 25.

*Honduran Records *(*14 Specimens*)

 ATLÁNTIDA: m2, f3 from *Artibeus lituratus*, Jardin Botanico Lancetilla, 12 July 2001; f2 from *A. lituratus*, Jardin Botanico Lancetilla, 14 July 2001.
 COMAYAGUA: m1 from *A. intermedius*, 4.8 km N, 8.7 km W Comayagua (Playitas), 10 July 2001; f2, f1 from *A. intermedius*, Siguatepeque, 18 July 2001.
 LEMPIRA: f1 from *Sturnira ludovici*, Parque Nacional Celaque, Centro de Visitantes, 11 October 1992, T. J. McCarthy, leg.; m1 from *S. ludovici*, Parque Nacional Celaque, Centro de Visitantes, 12 October 1992, T. J. McCarthy, leg.
 VALLE: f1 from *A. lituratus*, 13 km W, 3 km S Nacaome, 10 July 2001.

As in Venezuela [4], the characteristic host of *Paratrichobius longicrus* in Paraguay is *Artibeus lituratus* (hosting 156 of 159 specimens; 98.1%) [29]. Honduran records from hosts other than *A. lituratus* and *A. intermedius* are suspect. Simmons [24] considered *A. intermedius* a junior synonym of *A. literatus*. *Artibeus lituratus* is not known to harbor parasites of the genus *Megistopoda*, which are characteristic parasites of other species of *Artibeus* and *Sturnira* spp.*Paratrichobius* and *Megistopoda* may be ecological equivalents, being fairly similar in gross morphology with long hind legs. They differ, however, in that *Paratrichobius* spp. have fully functional wings while *Megistopoda* spp. have reduced and nonfunctional “strap-like” wings.

*Paratrichobius sp. *(*salvini complex*)

*Honduran Records *(*1 Specimen*)

 ATLÁNTIDA: m1 from *Platyrrhinus helleri* (Peters), Jardin Botanico Lancetilla, 12 July 2001.

*Paratrichobius salvini* was described from the host *Chiroderma salvini* Dobson [1]. Forms from *C. villosum* Peters, *C. trinitatum* Goodwin, *Platyrrhinus brachycephalus* (Rouk & Carter), and *P. helleri* are very similar to *Paratrichobius salvini* and were placed into the *P. salvini *species complex [4].

*Genus Speiseria Kessel, 1925*

 
*Speiseria* Kessel, 1925:19.

This genus comprises three described species: *Speiseria ambigua, S. magnioculus* Wenzel, and *S. peytonae *(Wenzel). *Speiseria ambigua* and *S. peytonae* are associated with bats of the genus *Carollia* (phyllostomid subfamily Carolliinae), while *S. magnioculus* is associated with *Trachops cirrhosus* (Spix) (phyllostomid subfamily Phyllostominae). Species are fully winged with long legs and are found in the furred regions of their hosts.

*Speiseria ambigua Kessel*

 
*Speiseria ambigua* Kessel, 1925:20, Figures 1-2, Plate 1.

*Honduran Records *(*9 Specimens*)

 ATLÁNTIDA: m3, f3 from *Carollia sowelli*, Jardin Botanico Lancetilla, 12 July 2001; f1 from *Mimon cozumelae* Goldman, Jardin Botanico Lancetilla, 12 July 2001; m2 from *C. sowelli*, Jardin Botanico Lancetilla, 15 July 2001.

In Venezuela, *Speiseria ambigua* is a parasite of *Carollia perspicillata*, as 96% of those collected in Venezuela were from 220 *C. perspicillata*. *Carollia brevicauda* is host to *S. peytonae*; the specimens from *C. sowelli* cannot be distinguished from *S. ambigua*.

*Speiseria peytonae Wenzel*

 
*Speiseria peytoni* Wenzel, 1976:127. Figure 52B (emended by Wenzel, 1984).

*Honduran Records *(*1 Specimen*)

 LEMPIRA: m1 from *Carollia brevicauda*, Parque Nacional Celaque, Centro de Visitantes, 17 February 1998, R. P. Eckerlin, leg.

Males of *S. peytonae* are easily distinguished from those of *S. ambigua* by the form of the genitalia.

*Genus Trichobioides Wenzel, 1966*

 
*Trichobioides* Wenzel, 1966:510.

This genus contains only one described species, *T. perspicillatus*.

*Trichobioides perspicillatus *(*Pessôa & Galvão*)

 
*Trichobioides perspicillatus* (Pessôa and Galvão) Wenzel, 1966:511, Figures 81, 82A.

*Honduran Records *(*37 Specimens*)

 ATLÁNTIDA: f1 from *Phyllostomus discolor*, Lancetilla, 6 August 1969, R. Valdez and R. K. LaVal, leg. ([20]; data compiled by author)
 COPÁN: m10, f8 from *P. discolor*, 20 km SE Santa Rosa de Copán, 17 July 2001.
 CORTÉS: m1 from *P. discolor*, Santo Domingo, 5.5 km ESE Cuyamel, 8 August 1988, T. J. McCarthy, leg.
 FRANCISCO MORAZON: m3, f1 from *P. discolor*, 12 mi N Tegucigalpa, no date, “GHJ”, leg. (Wenzel, [20] 1970; data compiled by author).
 VALLE: m3, f3 from *P. discolor*, 3 km N, 12.5 km SW San Lorenzo, 7 July 2001; m5, f2 from *P. discolor*, 13 km W, 3 km S Nacaome, 10 July 2001.

This species is a characteristic parasite of the phyllostomine bat *Phyllostomus discolor*. It is not known to occur on either of the other two species of *Phyllostomus*: *P. elongatus* (E. Geoffroy) and *P. hastatus*. All 37 Honduran specimens were collected from *P. discolor*, as were 97% of the 689 specimens collected in Venezuela [4].

*Genus Trichobius Gervais, 1844*

 
*Trichobius* Gervais, 1844:14.

The genus *Trichobius* currently comprises 68 described species and is the most diverse genus of the family Streblidae. The most recently described species were *T. machadoallisoni *Guerrero and *T. anducei* Guerrero [31]. Members of this genus parasitize a wide variety of bats, including representatives of the families Emballonuridae, Furipteridae, Molossidae, Mormoopidae, Natalidae, and Phyllostomidae. The genus has been divided into nine species groups or complexes [1, 4]: *pallidus* group (1 species), *caecus* group (7 species), *uniformis* group (4 species), *major* group (18 species), *longipes* group (8 species), *dugesii* group *dugesii *complex (12 species), *dugesii* group *parasiticus* complex (9 species), *phyllostomae *group (5 species), and the *dunni* group (4 species). Some of the described species of *Trichobius* are very distinct morphologically. Based on host associations and morphological affinities to other streblid genera, some workers feel that some if not all of the *Trichobius* species groups should be described as distinct genera (R. L. Wenzel, pers. comm.). The *T. phyllostomae* group has, based on morphological analysis, been shown to form a monophyletic group [32]. Yet other species are very similar morphologically and can be positively identified only by microslide mounting and examining under the high magnification of a compound microscope. The entire genus is in need of revision; it is predicted that the genus as currently constituted is widely paraphyletic. Thirteen species of *Trichobius* are known to occur in Honduras.

*Trichobius caecus Edwards*

 
*Trichobius caecus* Edwards, 1918:424.

*Honduran Records *(*62 Specimens*)

 ATLÁNTIDA: m3, f7 from *Pteronotus parnellii*, Jardin Botanico Lancetilla, 15 July 2001.
 COPÁN: m17, f21, 6 sex undetermined from *P. parnellii*, 20 km SE Santa Rosa de Copán, 17 July 2001; m2 from *Phyllostomus discolor*, 20 km SE Santa Rosa de Copán, 17 July 2001.
 OLANCHO: m5, f1 from *Pteronotus parnellii*, 4 km E Catacamas (Escuela de Sembrador), 20 July 2001.

*Trichobius caecus* is a member of the *caecus* group of species [4]. In Venezuela, 97% of the 1,592 collected specimens were taken from the mormoopid bat *P. parnellii*.

*Trichobius costalimai Guimarães*

 
*Trichobius costalimai* Guimarães, 1938:660, Figure 10, Plate 3.

*Honduran Records *(*75 Specimens*)

 ATLÁNTIDA: f2 from *Phyllostomus discolor*, Lancetilla, 6 August 1969, R. Valdez and R. K. LaVal, leg. ([20]; data compiled by author).
 COPÁN: m21, f10 from *P. discolor*, 20 km SE Santa Rosa de Copán, 17 July 2001.
 CORTÉS: m1 from *P. discolor*, Santo Domingo, 5.5 km ESE Cuyamel, 8 August 1988, T. J. McCarthy, leg.
 FRANCISCO MORAZON: m2, f1 from *P. discolor*, 12 mi N Tegucigalpa, no date, “GHJ”, leg. ([20; data compiled by author).
 VALLE: m5, f5, 1 sex undetermined from *P. discolor*, 3 km N, 12.5 km SW San Lorenzo, 7 July 2001; m16, f11 from *P. discolor*, 13 km W, 3 km S Nacaome, 10 July 2001.

*Trichobius costalimai* is a member of the *longipes* group of species [4]. In Honduras, this species was collected only from *Phyllostomus discolor*; in Venezuela, 96% of the 2,154 specimens collected were taken from *P. discolor*.

*Trichobius diphyllae Wenzel*

 
*Trichobius diphyllae* Wenzel, 1966: 492, Figures 68B, 73A.

*Honduran Records *(*2 Specimens*)

 ATLÁNTIDA: f2 from *Diphylla ecaudata*, Lancetilla, 9 August 1969, R. K. LaVal, leg.

*Trichobius diphyllae* is a member of the parasiticus complex of the *dugesii* species group [4].

*Trichobius dugesii Townsend*

 
*Trichobius dugesii* Townsend, 1891:106.

*Honduran Records *(*97 Specimens*)

 ATLÁNTIDA: f1 from *Mimon cozumelae*, Jardin Botanico Lancetilla, 12 July 2001; m2, f2 from *Glossophaga soricina*, Jardin Botanico Lancetilla, 13 July 2001; f1 from *G. soricina*, Jardin Botanico Lancetilla, 15 July 2001.
 COMAYAGUA: f1 from *G. soricina*, 4.8 km N, 8.7 km W Comayagua (Playitas), 9 July 2001; m10, f8 from *G. soricina*, 4.8 km N, 8.7 km W Comayagua (Playitas), 10 July 2001.
 LEMPIRA: m2, f2 from *G. commissarisi*, Parque Nacional Celaque, Centro de Visitantes, 11 October 1992, J. V. Planz, leg.; m1 from *G. soricina*, Parque Nacional Celaque, Centro de Visitantes, 13 October 1992, T. J. McCarthy, leg.
 OLANCHO: m3, f3 from *G. soricina*, 3 km N Catacamas (Escuela Nacional de Agricultura), 20 July 2001, C. W. Dick, leg.; f1 from *G. soricina*, 4 km E Catacamas (Escuela de Sembrador), 19 July 2001; m3 from *G. soricina*, 4 km E Catacamas (Escuela de Sembrador), 20 July 2001.
 VALLE: m1 from *G. commissarisi*, 3 km N, 12.5 km SW San Lorenzo, 7 July 2001; m1 from *G. leachii* Gray, 3 km N, 9 km SW San Lorenzo, 8 July 2001; m1, f1 from *G. soricina*, 3 km N, 9 km SW San Lorenzo, 8 July 2001; m1, f1 from *G. soricina*, 13 km W, 3 km S Nacaome, 9 July 2001.

*Trichobius dugesii* is a member of the *dugesii *complex of the *dugesii *group. The species appears to be stenoxenous, parasitizing several species of the glossophagine bat *Glossophaga*. In Honduras, it was collected from *G. commissarisi*, *G. leachii*, and *G. soricina*; in Venezuela, 97.7% of the 354 specimens collected were taken from *G. longirostris* Miller and *G. soricina*. The species cooccurs with a congener, *T. uniformis*.

*Trichobius galei Wenzel*

 
*Trichobius galei* Wenzel, 1966:449. Figures 57J–L.

*Honduran Records *(*7 Specimens*)

 FRANCISCO MORAZON: m3, f3 from *Natalus stramineus* Gray, Parque Nacional La Tigra, 6 July 2001.
 VALLE: m1 from *Glossophaga soricina*, 13 km W, 3 km S Nacaome, 9 July 2001.

*Trichobius galei* is a member of the *caecus *group of species [4]. Species of this group parasitize bats of the families Emballonuridae and Natalidae. In Panama [1] and Paraguay [29], *T. galei* was restricted to *N. stramineus*. In Venezuela, however, 98.5% of the 277 specimens collected were taken from the congener *N. tumidirostris* [4]. The Honduran record from *G. soricina* probably represents a contaminant.

*Trichobius hirsutulus Bequaert*

 
*Trichobius hirsutulus* Bequaert, 1933:572. Figures 30A, B.

*Honduran Records *(*10 Specimens*)

 CORTÉS: m5, f5 from *Myotis keaysi*, Santo Domingo, 5.5 km ESE Cuyamel, 8 August 1988, T. J. McCarthy, leg.

*Trichobius hirsutulus* is a member of the *major* group of species. The *major* group of species is the only true “northern” radiation of Streblidae; species are known from North America, Mexico, Central America, and Antilles, but none from farther south than Peru. Previous to this report, the species was known only from México (Tamaulipas and Yucatán), from the vespertilionid *Myotis nigricans* Schinz.

*Trichobius intermedius Peterson & Hůrka*

 
*Trichobius intermedius* Peterson & Hůrka, 1974:1049.

*Honduran Records *(*64 Specimens*)

 COMAYAGUA: m7, f3 from *Artibeus intermedius*, 4.8 km N, 8.7 km W Comayagua (Playitas), 10 July 2001; f2 from *Sturnira lilium*, 4.8 km N, 8.7 km W Comayagua (Playitas), 10 July 2001; m1, f1 from *Glossophaga commissarisi*, 4.8 km N, 8.7 km W Comayagua (Playitas), 10 July 2001.
 VALLE: m2, f1 from *A. lituratus*, 13 km W, 3 km S Nacaome, 10 July 2001; m25, f21 from *Artibeus inopinatus *Davis and Carter, 13 km W, 3 km S Nacaome, 9 July 2001.
 SIN DEPARTAMENTO: m1 from *A. jamaicensis*, “Tapasuna”, 1 December 1938, P. O. McGrew, leg. [21].

*Trichobius intermedius* is a member of the *dugesii* complex of the *dugesii *group of species. Throughout its range from Mexico, Antilles, to northern Central America, it is a characteristic parasite of large *Artibeus* species, particularly *A. jamaicensis*. Honduras appears to contain the terminus of its southern distribution. Extensive surveys have been undertaken in Costa Rica (C. W. Dick, R. M. Timm, R. L. Wenzel, unpublished data), Nicaragua (C. W. Dick, unpublished data), and Panama [1], but no *T. intermedius* were present in these collections.

*Trichobius joblingi Wenzel*

 
*Trichobius joblingi* Wenzel, 1966:481, Figures 68E, 70.

*Honduran Records *(*142 Specimens*)

 ATLÁNTIDA: m1 from *Carollia castanea* H. Allen, Jardin Botanico Lancetilla, 12 July 2001; m6, f1 from *C. perspicillata*, Jardin Botanico Lancetilla, 12 July 2001; m23, f13 from *C. sowelli*, Jardin Botanico Lancetilla, 12 July 2001; m17, f6 from *C. sowelli*, Jardin Botanico Lancetilla, 14 July 2001; m32, f24 from *C. sowelli*, Jardin Botanico Lancetilla, 15 July 2001; m1, f4 from *C. subrufa*, Jardin Botanico Lancetilla, 12 July 2001; m2, f2 from *C. subrufa*, Jardin Botanico Lancetilla, 15 July 2001.
 COMAYAGUA: m3 from *C. sowelli*, Cueva de Taulabe, 11 July 2001.
 LEMPIRA: m2 from *C. brevicauda*, Parque Nacional Celaque, Centro de Visitantes, 11 October 1992, J. V. Planz, leg.; m2 from *C. brevicauda*, Parque Nacional Celaque, Centro de Visitantes, 17 February 1998, R. P. Eckerlin, leg.
 OLANCHO: m3 from *C. sowelli*, 4 km E Catacamas (Escuela de Sembrador), 20 July 2001.

*Trichobius joblingi* is a member of the *dugesii* complex of the *dugesii* complex of species. The species is a characteristic parasite of *C. perspicillata* throughout the extent of its range.

*Trichobius longipes Rudow*

 
*Trichobius longipes* Rudow, 1871:121.

*Honduran Record *(*3 Specimens*)

 ATLÁNTIDA: m2 from *Phyllostomus hastatus*, Lancetilla, 9 August 1969, R. Valdez & R. K. LaVal, leg.
 CORTÉS: m1 from *P. hastatus*, Omoa, Fortaleza de San Francisco de Omoa, 6 August 1988, T. J. McCarthy, leg.

*Trichobius parasiticus Gervais*

 
*Trichobius parasiticus* Gervais, 1844:14, Plate 43.

*Honduran Records *(*858 Specimens*)

 ATLÁNTIDA: m125, f82 from *Desmodus rotundus*, Jardin Botanico Lancetilla, 12 July 2001; m12, f9 from *D. rotundus*, Jardin Botanico Lancetilla, 13 July 2001; m255, f268 from *D. rotundus*, Jardin Botanico Lancetilla, 15 July 2001; m7, f3 from *Pteronotus parnellii*, Jardin Botanico Lancetilla, 15 July 2001.
 COMAYAGUA: m1 from *Artibeus intermedius*, Siguatepeque, 18 July 2001.
 COPÁN: f1 from *Phyllostomus discolor*, 20 km SE Santa Rosa de Copán, 17 July 2001.
 FRANCISCO MORAZON: m21, f17 from *D. rotundus*, Parque Nacional La Tigra, 6 July 2001.
 LEMPIRA: m5, f2, 1 sex undetermined from *D. rotundus*, 3.5 mi N Gracias, 8 January 1938, P. O. McGrew, leg. ([20]; data compiled by author); m2, f3 from *D. rotundus*, Parque Nacional Celaque, Centro de Visitantes, 11 October 1992, J. V. Planz, leg.; m7, f4 from *D. rotundus*, Parque Nacional Celaque, Centro de Visitantes, 12 October 1992, S. R. Flores, leg.; m11, f14 from *D. rotundus*, Parque Nacional Celaque, Centro de Visitantes, 17 February 1998, R. P. Eckerlin, leg.
 OLANCHO: m1, f2 from *Rhynchonycteris naso* (Wied-Neuwied), 4 km E Catacamas (Escuela de Sembrador), 19 July 2001.
 VALLE: m4 from *D. rotundus*, 13 km W, 3 km S Nacaome, 9 July 2001.

*Trichobius parasiticus* is a characteristic parasite of the common vampire bat, *Desmodus rotundus*. Wenzel et al. [1] reported that on *D. rotundus*, *T. furmani* replaces *T. parasiticus* in some parts of South America.

*Trichobius sparsus Kessel, 1925*

 
*Trichobius sparsus* Kessell, 1925:17. Figures 7, 10.

*Honduran Records *(*16 Specimens*)

 ATLÁNTIDA: m3, f2 from *Pteronotus parnellii*, Jardin Botanico Lancetilla, 15 July 2001; m6, f5 from *Carollia sowelli*, Jardin Botanico Lancetilla, 15 July 2001.

The association with *C. sowelli* is suspect; in Venezuela, all but one of 112 specimens were associated with *P. parnellii* [4]. The Jardin Botanico specimens were found on one bat of each species previously stated, captured at the same time in the same mist net.

*Trichobius uniformis Curran*

 
*Trichobius uniformis* Curran, 1935:10, Figure 8.

*Honduran Records *(*37 Specimens*)

 ATLÁNTIDA: f1 from *Artibeus lituratus*, Jardin Botanico Lancetilla, 12 July 2001; f1 from *Mimon cozumelae*, Jardin Botanico Lancetilla, 12 July 2001; m7, f1 from *Glossophaga soricina*, Jardin Botanico Lancetilla, 13 July 2001; m6, f2 from *G. soricina*, Jardin Botanico Lancetilla, 15 July 2001.
 COMAYAGUA: f1 from *G. soricina*, 4.8 km N, 8.7 km W Comayagua (Playitas), 9 July 2001; m1 from *G. commissarisi*, 4.8 km N, 8.7 km W Comayagua (Playitas), 10 July 2001; f3 from *G. soricina*, 4.8 km N, 8.7 km W Comayagua (Playitas), 10 July 2001.
 LEMPIRA: m1, f1 from *G. commissarisi*, Parque Nacional Celaque, Centro de Visitantes, 11 October 1992, J. V. Planz, leg.
 OLANCHO: m3, f5 from *G. soricina*, 3 km N Catacamas (Escuela Nacional de Agricultura), 20 July 2001; m2, f1 from *G. soricina*, 4 km E Catacamas (Escuela de Sembrador), 20 July 2001.
 VALLE: m1 from *G. soricina*, 13 km W, 3 km S Nacaome, 9 July 2001.

This bat fly cooccurs on *G. soricina* with another congener, *T. dugesii*.

*Trichobius Undescribed Species from Tonatia saurophila*

*Honduran Records *(*5 Specimens*)

 ATLÁNTIDA: m4, f1 from *Tonatia saurophila*, Jardin Botanico Lancetilla, 12 July 2001.

The existence of an undescribed species of *Trichobius* from the bat *Tonatia saurophila* was first noted by Wenzel (pers. comm.) and later by Miller and Tschapka [30]. Because a long series of specimens are known from Costa Rica (ca. 50 specimens), this species will be described elsewhere, in a treatment of the Costa Rican fauna.

## 4. Discussion

This paper provides the most comprehensive treatment to date of streblid bat flies known from Honduras and adds 11 genera (65%) and 32 species (74%) to the known Honduran fauna. From this treatment of a small but biologically important family of Diptera, it is clear that a great need exists in Honduras for systematic biodiversity surveys, during which museum specimens are prepared and identified and the rich fauna of Honduras is more fully described and explained. The need for baseline information on Honduran Streblidae cannot be overemphasized. At this time, 17 genera and 43 described species of streblid bat flies have been documented to occur in Honduras.

Streblid species richness in Honduras compares relatively well to other neotropical localities. Although relatively few comprehensive treatments of streblid flies exist for neotropical countries, those that do exist are insightful. In Panama, approximately 100 bat species were sampled yielding 66 fly species [1]. In Venezuela, approximately 130 bat species were sampled yielding 115 fly species [4]. In Paraguay, 54 bat species were sampled, yielding 31 fly species [29]. In the present study, approximately 45 species of bats were sampled, yielding 43 fly species. These trends in fly species richness relative to host species richness comport previous findings of positive correlation between fly and host richness values [29]. A relatively comprehensive treatment of Guatemalan bat flies yielded a fly species richness of 40 species, comparable to Honduras. However, the number of bats sampled for the Guatemalan collection is unknown [22].

The International Union for Conservation of Nature [33] lists 96 species of Chiroptera for Honduras. This is nearly identical to the 95 bat species reported for Guatemala [34]. Given the number of bat species in Honduras and assuming (1) that each of these is in fact host to fly species known from those hosts from other neotropical localities and (2) that each bat species is parasitized by unique species of Streblidae (e.g., [2, 3, 35]), it is possible to estimate the streblid bat fly species potentially in Honduras. Following neotropical host-parasite associations summarized in [4, 23], I estimate that the number of species in Honduras may be up to four times higher (ca. 170 spp.) than the number reported here.

Future work must be conducted in Honduras, particularly work that involves the collection of bats and their ectoparasites. Even simple specimen collection surveys would facilitate reasonable estimates of Honduras' biodiversity. Although Honduras is incredibly rich in biodiversity, knowledge of this biodiversity is little developed. Particularly in the light of pressure to modify natural habitat to suit ever expanding material needs of humankind, the time is right to make biodiversity surveys of Honduras, in order to assess and conserve its unique and important biodiversity.

## Figures and Tables

**Table 1:** Gazetteer of Honduran bat fly collection localities. Locality numbers correspond to map (Figure 1). Latitude and longitude are in decimal degrees. Specific localities with identical locality numbers are close in proximity, not warranting separate placement on the map (Figure 1).

Department	Locality	Specific locality	Lat	Long
Atlántida	1	Jardin Botanico Lancetilla	15.7404	−87.4572
Atlántida	1	Jardin Botanico Lancetilla (net 1)	15.7457	−87.4541
Atlántida	1	Jardin Botanico Lancetilla (net 2)	15.7388	−87.4552
Atlántida	2	Lancetilla	15.6964	−87.4319
Comayagua	3	Comayagua (Senasa)	14.4551	−87.6569
Comayagua	4	Cueva de Taulabe	14.6949	−87.9519
Comayagua	5	Siguatepeque	14.5902	−87.8600
Comayagua	6	4.8 km N, 8.7 km W Comayagua (Playitas)	14.4280	−87.7023
Copán	7	0.5 km N, 1 km W Santa Rosa de Copán	14.7880	−88.7841
Copán	8	20 km SE Santa Rosa de Copán	14.7682	−88.6926
Copán	9	5 km NW Santa Rosa de Copán	14.8532	−88.7688
Cortés	10	Omoa, Fortaleza de San Francisco de Omoa	15.5079	−88.0355
Cortés	11	Santo Domingo, Sierra Omoa, ~5.5 km ESE Cuyamel	15.6587	−88.1439
Francisco Morazán	12	Parque Nacional La Tigra	14.2056	−87.1160
Francisco Morazán	13	Parque Zoologia El Picacho	14.1225	−87.0236
Francisco Morazán	14	12 mi N Tegucigalpa	14.2044	−87.2156
Lempira	15	Parque Nacional Celaque, Centro de Visitantes	14.5500	−88.6333
Lempira	15	Parque Nacional Celaque, Don Tomas	14.5333	−88.6500
Lempira	16	3.5 mi N Gracias	14.6506	−88.5827
Olancho	17	Danli: 78 mi ENE by E Rio Coco	14.4652	−85.6576
Olancho	18	Parque Nacional Agalta, Sendero a la Picucha	15.0124	−85.8615
Olancho	19	Parque Nacional de La Muralla, Los Cuatro Pavos	15.0983	−86.7333
Olancho	20	3 km N Catacamas (Escuela Nacional de Agricultura)	14.8258	−85.8450
Olancho	20	4 km E Catacamas (Escuela de Sembrador)	14.8088	−85.8428
Islas de la Bahía	21	Isla Roatán, Coxen Hole	16.3293	−86.5303
Valle	22	13 km W, 3 km S Nacaome	13.5152	−87.5965
Valle	23	3 km N, 12.5 km SW San Lorenzo	13.4240	−87.5446
Valle	24	3 km N, 9 km SW San Lorenzo	13.4484	−87.5270

## Appendices

### A. Concise List of the 17 Genera and 43 Species of Streblid Bat Flies Known to Occur in Honduras

New records (11 genera and 32 species) for Honduras are indicated by an asterisk as follows:

**Anastrebla modestini* Wenzel, 1966
*Anatrichobius scorzai* Wenzel, 1966
**Aspidoptera falcata* Wenzel, 1976
**Aspidoptera phyllostomatis* (Perty), 1833
*Exastinion clovisi* (Pessôa & Guimarães), 1937
**Mastoptera guimaraesi* Wenzel, 1966
**Mastoptera minuta* (Costa Lima), 1921
**Mastoptera* sp. (minuta complex)
*Megistopoda aranea* (Coquillett), 1899
*Megistopoda proxima* (Séguy), 1926
**Megistopoda theodori* Wenzel, 1966
**Metelasmus pseudopterus* Coquillett, 1907
**Neotrichobius stenopterus* Wenzel & Aitken, 1966
**Noctiliostrebla traubi* Wenzel, 1966
**Nycterophilia coxata* Ferris, 1916
**Paradyschiria fusca* Speiser, 1900
**Paradyschiria parvuloides* Wenzel, 1966
**Paraeuctenodes longipes* Pessôa & Guimarães, 1937
**Paratrichobius dunni* (Curran), 1935
**Paratrichobius longicrus* (Miranda Ribeiro), 1907
**Paratrichobius* sp. (*salvini* complex)
**Speiseria ambigua* Kessel, 1925
**Speiseria peytonae* Wenzel, 1976
**Strebla chrotopteri* Wenzel, 1976
**Strebla curvata* Wenzel, 1976
**Strebla diphyllae* Wenzel, 1966
**Strebla galindoi* Wenzel, 1966
*Strebla guajiro* (García & Casal), 1965
*Strebla hertigi* Wenzel, 1966
**Strebla kohlsi* Wenzel, 1966
*Strebla wiedemanni* Kolenati, 1856
*Trichobioides perspicillatus* (Pessôa & Galvão), 1937
**Trichobius caecus* Edwards, 1918
*Trichobius costalimai* Guimarães, 1938
**Trichobius diphyllae* Wenzel, 1966
**Trichobius dugesii* Townsend, 1891
**Trichobius galei* Wenzel, 1966
**Trichobius hirsutulus* Bequaert, 1933
*Trichobius intermedius* Peterson & Hurka, 1974
**Trichobius joblingi* Wenzel, 1966
**Trichobius longipes* (Rudow), 1871
*Trichobius parasiticus* Gervais, 1844
**Trichobius sparsus* Kessel, 1925
**Trichobius uniformis* Curran, 1935
**Trichobius* undescribed species (*dugesii* group) from *Tonatia saurophila*.

### B. List of 43 Streblid Bat Fly Species Known from Honduras, with Bat Host Associations

Numbers in parentheses indicate (1) the number of individual flies collected of that fly species and on that host species and (2) the percentage of the total fly individuals collected that were associated with that host species. Associations noted by asterisks are generally considered accidental or contamination transfers and are determined by reference to general patterns of host association among neotropical streblid flies [1, 2, 4, 29]; refer to species accounts for additional information:

*Anastrebla modestini *(1)
  *Glossophaga soricina* (1) (100%)
*Anatrichobius scorzai *(6 specimens)
  *Myotis keaysi* (6) (100%)
*Aspidoptera falcata *(39)
  *Sturnira lilium* (23) (60%)
  *S. ludovici* (15) (38.5%)
  **Noctilio leporinus* (1) (2.5%)
*Aspidoptera phyllostomatis *(14)
  *Artibeus jamaicensis* (10) (71.4%)
  **A. lituratus* (3) (21.4%)
  **Glossophaga soricina* (1) (7.1%)
*Exastinion clovisi *(5)
  *Anoura geoffroyi* (5) (100%)
*Mastoptera guimaraesi *(3)
  *Phyllostomus hastatus* (3) (100%)
*Mastoptera minuta *(5)
  *Lophostoma silvicolum* (5) (100%)
*Mastoptera* sp.  (23)
  *Lophostoma brasiliense* (23) (100%)
*Megistopoda aranea *(31)
  *Artibeus jamaicensis* (22) (71%)
  *A. intermedius* (7) (22.6%)
  **A. lituratus* (1) (3.2%)
  **Glossophaga soricina* (1) (3.2%)
*Megistopoda proxima *(58)
  *Sturnira lilium* (50) (86.2%)
  **Artibeus lituratus* (3) (5.2%)
  **Glossophaga commisarisi* (3) (5.2%)
  **G. soricina* (1) (1.7%)
  **Dermanura phaeotis* (1) (1.7%)
*Megistopoda theodori *(52)
  *Sturnira ludovici* (51) (98.1%)
  **Uroderma bilobatum* (1) (1.9%)
*Metelasmus pseudopterus *(4)
  *Artibeus jamaicensis* (2) (50%)
  *A. lituratus* (1) (25%)
  **Carollia sowelli* (1) (25%)
*Neotrichobius stenopterus *(3)
  *Dermanura phaeotis* (2) (67%)
  *D. toltecus* (1) (33%)
*Noctiliostrebla traubi *(34)
  *Noctilio leporinus* (34) (100%)
*Nycterophilia coxata *(362)
  *Pteronotus parnellii* (361) (99.7%)
  **Sturnira lilium* (1) (0.3%)
*Paradyschiria fusca *(43)
  *Noctilio leporinus* (43) (100%)
*Paradyschiria parvuloides *(10)
  *Noctilio albiventris* (10) (100%)
*Paraeuctenodes longipes *(3)
  *Glossophaga commisarisi* (2) (67%)
  *G. soricina* (1) (33%)
*Paratrichobius dunni *(3)
  *Uroderma bilobatum* (2) (67%)
  **Dermanura phaeotis* (1) (33%)
*Paratrichobius longicrus *(14)
  *Artibeus lituratus* (8) (57%)
  **A. intermedius* (4) (29%)
  **Sturnira ludovici* (2) (14%)
*Paratrichobius* sp. (*salvini complex*)  (1)
  *Platyrrhinus helleri* (1) (100%)
*Speiseria ambigua *(9)
  *Carollia sowelli* (8) (89%)
  **Mimon cozumelae* (1) (11%)
*Speiseria peytonae *(1)
  *Carolllia brevicauda* (1) (100%)
*Strebla chrotopteri *(5)
  *Chrotopterus auritus* (5) (100%)
*Strebla curvata *(7)
  *Glossophaga soricina* (6) (86%)
  **Artibeus lituratus* (1) (14%)
*Strebla diphyllae *(2)
  *Diphylla ecaudata* (2) (100%)
*Strebla galindoi *(1)
  *Tonatia saurophila* (1) (100%)
*Strebla guajiro *(13)
  *Carollia sowelli* (6) (46%)
  **C. brevicauda* (3) (23%)
  *C. perspicillata* (1) (7.7%)
  **Glossophaga soricina* (1) (7.7%)
  **Artibeus lituratus* (1) (7.7%)
  *unknown host (1) (7.7%)
*Strebla hertigi *(5)
  *Phyllostomus discolor* (5) (100%)
*Strebla kohlsi *(5)
  *Lophostoma silvicolum* (5) (100%)
*Strebla wiedemanni *(69)
  *Desmodus rotundus* (69) (100%)
*Trichobioides perspicillatus *(37)
  *Phyllostomus discolor* (37) (100%)
*Trichobius caecus *(62)
  *Pteronotus parnellii* (60) (97%)
  **Phyllostomus discolor* (2) (3%)
*Trichobius costalimai *(75)
  *Phyllostomus discolor* (75) (100%)
*Trichobius diphyllae *(2)
  *Diphylla ecaudata* (2) (100%)
*Trichobius dugesii *(97)
  *Glossophaga soricina* (90) (93%)
  *G. commissarisi* (5) (5%)
  **G. leachii* (1) (1%)
  **Mimon cozumelae* (1) (1%)
*Trichobius galei *(7)
  *Natalus stramineus* (6) (86%)
  **Glossophaga soricina* (1) (14%)
*Trichobius hirsutulus *(1)
  *Myotis keays*i (1) (100%)
*Trichobius intermedius *(64)
  *Artibeus inopinatus* (46) (72%)
  *A. intermedius* (10) (16%)
  **A. lituratus* (3) (5%)
  **Glossophaga commissarisi* (2) (3%)
  **Sturnira lilium* (2) (3%)
  *A. jamaicensis* (1) (2%)
*Trichobius joblingi *(142)
  *Carollia sowelli* (121) (85%)
  *C. subrufa* (9) (6%)
  *C. perspicillata* (7) (5%)
  *C. brevicauda* (4) (3%)
  **C. castanea* (1) (1%)
*Trichobius longipes *(3)
  *Phyllostomus hastatus* (3) (100%)
*Trichobius parasiticus *(857)
  *Desmodus rotundus* (842) (98.3%)
  **Pteronotus parnellii* (10) (1.2%)
  **Rhynchonycteris naso* (3) (0.1%)
  **Phyllostomus discolor* (1) (0.1%)
  **Artibeus intermedius* (1) (0.1%)
*Trichobius sparsus *(16)
  **Carollia sowelli* (11) (69%)
  *Pteronotus parnellii* (5) (31%)
*Trichobius uniformis *(37)
  *Glossophaga soricina* (29) (78%)
  *G. commissarisi* (6) (16%)
  **Artibeus lituratus* (1) (3%)
  **Mimon cozumelae* (1) (3%)
*Trichobius* undescribed species (*dugesii* group)  (5)
  *Tonatia saurophila* (5) (100%).
